# Systematic literature review on the safety and immunogenicity of rotavirus vaccines when co-administered with meningococcal vaccines

**DOI:** 10.1080/21645515.2020.1739485

**Published:** 2020-04-16

**Authors:** Priya Pereira, Bernd Benninghoff, Leentje Moerman

**Affiliations:** GSK Vaccines, Wavre, Belgium

**Keywords:** Rotavirus, *Neisseria meningitidis*, meningococcal vaccines, co-administration, safety, immunogenicity, infants, systematic literature review

## Abstract

This study is aimed to review the published evidence on safety, immunogenicity, and efficacy of rotavirus vaccines when co-administered with meningococcal vaccines in infants. A systematic literature search was performed in four databases containing peer-reviewed articles and conference abstracts. In total, twelve articles were included in the review; 11 provided information on safety and five on the immunogenicity of rotavirus vaccines following co-administration. No paper was found on efficacy. Additional routine vaccines were administered in all studies. The safety analysis was mainly focused on fever, vomiting, diarrhea, intussusception, and changes in eating habits. Overall, safety profiles and immune responses associated with rotavirus vaccination were comparable between infants co-administered with rotavirus and meningococcal vaccines and infants receiving rotavirus vaccines without meningococcal vaccines. Although data are limited, co-administration of rotavirus and meningococcal vaccines does not appear to interfere with the safety or immunogenicity of rotavirus vaccines.

## Introduction

Rotavirus (RV) is a common cause of severe and fatal acute diarrhea in young children throughout the world.^1^ In the European Union, 300–600 children per 100,000 under the age of 5 years are hospitalized due to RV gastroenteritis (RVGE) annually.^2^ Many countries worldwide have already included oral RV vaccines in their National Immunization Programs (NIP) as recommended by the World Health Organization (WHO).^3,4^ Following RV vaccine introduction, significant reduction in infant diarrheal deaths, RVGE-related hospitalization, and cases of RVGE was observed.^1^ Currently, there are two globally available oral vaccines for the prevention of RVGE in the first year of life. The human live-attenuated RV vaccine containing G1P[8] strain (HRV, *Rotarix*) is administered as a two-dose schedule, with a minimum interval of 4 weeks between doses, the first dose being administered from 6 weeks of age. The schedule must be completed by 24 weeks of age.^5,6^ The human-bovine live-attenuated reassortant pentavalent RV vaccine (HBRV, *RotaTeq*) is administered as a three-dose regimen, with an interval of at least 4 weeks between doses, the first dose being administered between 6 and 12 weeks of age. The schedule must be completed by 32 weeks of age.^7,8^ Additionally, new live-attenuated naturally reassorted monovalent (nHRV, *Rotavac*) and human-bovine reassortant pentavalent (BRV-PV, *Rotasiil*) RV vaccines have been recently licensed.^9,10^ All these four vaccines are now WHO prequalified.^11^

Invasive meningococcal disease (IMD), characterized by high mortality and morbidity, is mainly caused by six (MenA, MenB, MenC, MenW, MenY, and MenX) of the 12 serogroups of *Neisseria meningitidis*. Epidemiology of IMD varies considerably geographically, with socio-economic setting, and over time.^12^ Different meningococcal vaccines are available to prevent IMD. Following recommendations of these vaccines in immunization programs, disease incidences decreased significantly in many countries worldwide.^13,14^ In young children, meningococcal vaccines are generally administered from 2 to 24 months of age: recombinant four-component MenB vaccine (4CMenB, *Bexsero*), Hib and MenC (*Menitorix*) and MenC and MenY (Hib-MenCY-TT, *MenHibrix*, discontinued in 2016) tetanus toxoid conjugated vaccines, tetravalent MenA, MenC, MenW-135, MenY diphtheria toxin (MenACWY-CRM, *Menveo*, and *Menactra*) and tetanus toxin conjugated vaccines (*Nimenrix*), MenA (MenAV, *MenAfriVac*) and MenC (MenCC, *NeisVac-C*) tetanus toxoid conjugated vaccines, and MenC diphtheria toxin conjugated vaccines (MenC-CRM, *Meningitec*, and *Menjugate*).^15^

Since the vaccination schedules for RV and some meningococcal vaccines overlap, co-administration of these vaccines as part of the routine immunization is likely.^16,17^ Given the increased recommendations of meningococcal vaccines worldwide, a summary of data on the safety, immunogenicity, and efficacy of their co-administration with RV vaccines is of interest to demonstrate the compatibility of these vaccines. This evidence could more easily prompt healthcare practitioners to boost parental attitude toward vaccination and to co-administer the vaccines, thus increasing RV and meningococcal vaccine coverage amongst children <5 year-olds, while saving on costs and number of medical visits required to comply with the routine vaccination schedule.^18^

## Methods

The objectives of this systematic literature search were to identify the published worldwide evidence on the safety profile, immunogenicity, and efficacy of RV vaccines when co-administered (at the same clinical visit) with meningococcal vaccines in infants during the first year of life.

## Identification, selection, and data extraction

The literature search was carried out in PubMed, Embase, Cochrane Library, and LILACS (Latin America) databases for peer-reviewed articles published between 1 January 2000 and 04 January (PubMed), 11 January (Embase), 13 February (Cochrane and LILACS) 2019. In addition, international conference abstracts published between 2014 and 2018, selected from the hits from the Embase search, were searched to identify studies not yet published in peer-reviewed journals. A gray literature search (03 March 2019) was also conducted to identify data for the remaining gaps. The websites of Pan American Health Organization (PAHO), Centers for Disease Control and Prevention (CDC), European Center for Disease Prevention and Control (ECDC), WHO, and regional agencies (National Health Service England and Ministries of Health of Spain, Brazil, and Italy) were searched for relevant data. In addition, an internet search was performed using the terms “rotavirus vaccines,” “meningococcal vaccines,” and “coadministration.” Search strings are provided in Supplementary Table S1.

Relevant studies were selected by a three-step selection procedure checking against a list of inclusion and exclusion criteria (Table 1). In a first step, a screening of titles and abstracts was performed in duplicate by two epidemiologists and relevant papers were selected for full-text screening. In case of doubt, the article was included in the full-text screening step. In a second step, full-text papers were screened in duplicate by two epidemiologists. Critical appraisal of full-text articles was performed only if the article provided information for one of the review objectives. Further scrutiny of articles was carried out during the data-extraction step (third step) to identify any additional information that might be relevant to be included in the current assessment. The reference lists of selected articles, narratives, and systematic reviews were checked manually for potentially relevant studies. Data from the selected articles were summarized using a standardized data-extraction spreadsheet in Excel. If needed, percentages of safety and immunogenicity parameters were calculated from the original data. If the article referred to a registration number at clinicaltrials.gov, the website was checked and relevant data were extracted.

**Table 1.** Systematic literature review: inclusion and exclusion criteria during the selection phases

	Inclusion	Exclusion
Period of publication	2000 onwards	Conference abstracts published before 2014
Study design/type	Studies providing original data	Systematic reviews and meta-analysis* Narrative reviews* Animal studies Case studies Articles that describe non-pertinent publication types (e.g. letters to the editor, editorials or comments)
Study population	Children <12 months old	Non-pertinent age groups (adolescents, adults)
Study comparison	Co-administration of rotavirus vaccine and meningococcal vaccine; Rotarix 2 dose vaccine schedule is from 6–24 weeks with minimum duration of 4 weeks between doses.	Clearly no co-administration of rotavirus vaccine and meningococcal vaccine
Study data	Quantitative and qualitative outcomes of all types of Rotavirus vaccination (immunogenicity, efficacy and/or safety data)	Data not relevant for the objectives (e.g. data of meningococcal vaccination outcomes)

* Reference lists were checked on possibly missed relevant articles. If this was the case, the original articles were included and the systematic review or meta-analysis itself was excluded.

## Study quality assessment

The original research articles eligible for data extraction were assessed using the SIGN checklists; this included criteria of the Cochrane checklists and the most important criteria of PRISMA and STROBE guidelines.^19^ The studies were rated as “high” quality if there was little or no risk of bias and the results were not likely to be changed by further research, “acceptable” quality if some flaws in the study were present with an associated medium risk of bias and results may change in the light of further studies, and “low” quality if significant flaws associated with high risk of bias were present. The final decision whether the quality of a study was sufficient or not for inclusion was based on the expertise of one epidemiologist, keeping the results of the checklist and the objectives of the review in mind. In case of doubt, the methodological quality of the study was discussed with the second epidemiologist.

## Results

**Figure 1.**
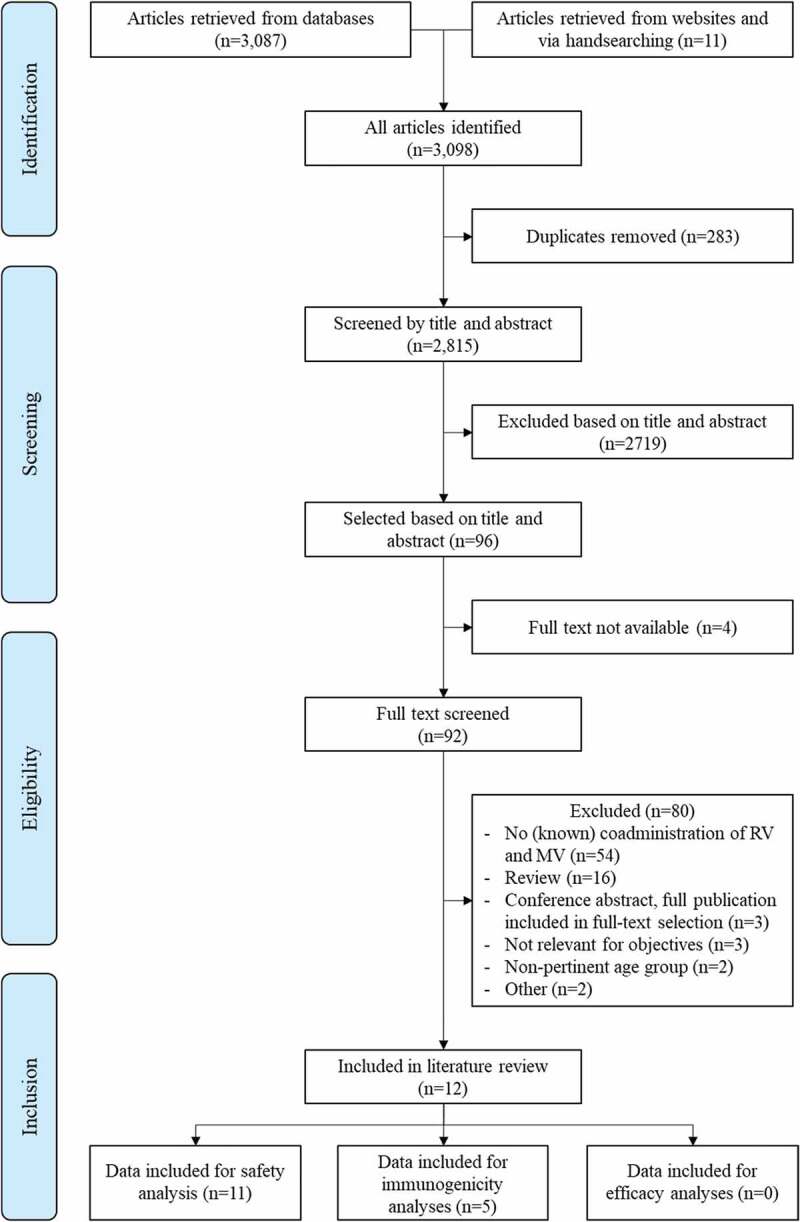
Selection procedure

RV: Rotavirus vaccine; MV: meningococcal vaccine.

In total, 3,098 references (including 342 conference abstracts) were retrieved from the databases and websites or identified by manual searching. Of the 2,815 unique records, twelve articles were finally included in the review (Figure 1). No additional articles were found through the gray literature search. Study design and characteristics are presented in Table 2. Quality of selected studies was mostly “acceptable.” After the initial screening, seven conference abstracts were selected for in-depth analysis. Three eligible abstracts referred to data published in full reports; all three reports were added to the full-text screening. The remaining four abstracts were considered not relevant with respect to objectives or did not report data on co-administration of RV and meningococcal vaccines. Eleven studies provided information on the safety profile of co-administration of RV vaccines and meningococcal vaccines^20-30^ and five studies reported data on immunogenicity.^21,22,24,29,31^ No studies presented data on vaccine efficacy.

**Table 2.** Description of studies included in the literature review

	Country	Study design	Study period	Study population	Sample size	Related vaccines	Schedules		Study quality
							Co-administration group	Control group	
Block 2016^27^ (NCT01214837)	United States and Canada	Open-label, randomized study	Oct 2010–May 2012	Healthy infants aged 55–89 days	733	RV: NR MV: MenACWY-CRM	RV: part of routine vaccinations: given at 2, 4 (and 12) MoA or 2, 4, 6 (and 12) MoA MV: at 2,4 (and 12) MoA or MV at 2, 4, 6 (and 12) MoA. RV. Schedule not clear	RV: part of routine vaccinations: given at 2, 4 (and 12) MoA or 2, 4, 6 (and 12) MoA. Schedule not clear	Acceptable
Bryan 2018^30^	United Kingdom	Prospective surveillance study	Sept 2015–May 2017	Infants aged between 8 weeks and <18 months.	Approx. 1,290,000	RV: probably HRV MV: 4CMenB	RV and MV combined at 8 WoA; MV at 16 WoA + routine vaccines	NA	Low
Haidara 2018^21^ (NCT02286895)	Mali	Open-label individual-randomized trial	Oct 2014–March 2015	Healthy children aged 9–11 months	600	RV: HBRV MV: MenAV	Booster dose of RV and MV + other vaccines	MV + other vaccines	Acceptable
Klein 2012^28^ (NCT00474526)	United States	Open-label randomized parallel-group multicenter study	March 2007–Nov 2009	Healthy infants aged 55–89 days, born ≥37 weeks and birth weight ≥2.5 kg, that received at least one vaccine dose	1,364	RV: HBRV MV: MenACWY-CRM	RV and MV combined at 2, 4, and 6 MoA + routine vaccines	RV alone (followed by MV in the second year of life) at 2, 4 and 6 MoA + routine vaccines	Acceptable
Klein 2019^22^ (NCT01978093)	United States	Randomized open-label multicentre trial	Feb 2014–March 2016	Healthy infants aged 6–12 weeks born ≥37 weeks	600	RV: HRV MV: Hib-MenCY-TT	RV and MV combined at 2 and 4 MoA, MV alone at 6 and 12–15 MoA + routine vaccines	RV at 2 and 4 MoA + routine vaccines	Acceptable
Martinon-Torres 2017^20^ (NCT01839188)	Spain	Open-label single armed multicenter study	March 2013–March 2014	Healthy infants aged 46–74 days at enrollment and received 1 dose of HepB vaccine ≤3 days of birth	384	RV: HBRV MV: MenCC	RV and MV combined and 2 and 4 MoA. RV at 6 MoA + routine vaccines	NA	Acceptable
Martinon-Torres 2019^24^ (U1111-1122–2329 and U1111-1122–2362)*	Spain	Two consecutive open-label studies (primary and booster vaccination)	Jan 2014–Nov 2015	Healthy infants aged 55–75 days, born ≥37 weeks, birth weight ≥2.5 kg	263	RV: HBRV MV: MenCC	RV and MV at 2 MoA, RV at 4 and 6 MoA + routine vaccines	NA	Acceptable
O’Ryan 2014^23^ (Review article)	Worldwide	NA	NA	Infants, adolescents, and adults	5,515 (>7000 including toddlers)	RV: HRV or HBRV MV: 4CMenB	According to the corresponding study design.	NA	Low
Tregnagh 2014^26^ (NCT00474526)	Argentina and Colombia	Open-label randomized trial	March 2007–Nov 2019	Healthy infants aged 55–89 days old, born ≥37 weeks and birth weight ≥2.5 kg, that received at least one vaccine dose	3,033	RV: HBRV MV: MenACWY-CRM	RV and MV at 2, 4 and 6 MoA + routine vaccines	RV at 2, 4 and 6 MoA (and MV toddler dose at 12 and 15 MoA) + routine vaccines	Acceptable
Vesikari 2011^29^ (NCT00443846)	Finland	Randomized trial	Jan 2007–March/2007	Healthy infants aged 6–7 weeks	247	RV: HBRV MV: MenCC	RV and MV combined at 10–11 WoA; RV and MV combined at 20–21 WoA; RV alone at 24–25 WoA + routine vaccines	RV at 6–7 WoA; MV at 10–11 WoA; RV at 15–16 WoA; MV at 20–21 WoA; RV at 24–25 WoA + routine vaccines	Acceptable
Vesikari 2010^31^ (NCT00140686)	Czech Republic, Finland, France, Germany, Italy, Spain	Randomized controlled trial	Sept 2004–Aug 2006	Healthy infants aged 6–7 weeks and birth weight >2000 g	1,216	RV: HRV MV: MenC-CRM (Spain only)	RV and MV combined at 2 and 4 MoA. An additional dose of vaccines was provided at 6 MoA (not clear what type). + routine vaccines	RV at 2 and 3–4 MoA (Germany, France), at 4 and 5 MoA (Finland and Italy), at 3 and 4 MoA (Czech Republic). + routine vaccines	Acceptable
Vesikari 2017^25^ (NCT01839175)	Finland	Randomized controlled multicenter trial	April 2012–Feb 2015	Healthy infants aged ≥46 days and ≤74 days, born ≥37 weeks and/or with birth weight ≥2.5 kg	350	RV: HBRV MV: MenCC	RV and MV combined at 2 and 4 MoA and RV alone at 3 MoA + routine vaccines	RV at 2,3, 4 MoA + routine vaccines	Acceptable

RV: rotavirus vaccines; MV: meningococcal vaccines; NR: not reported; NA: not applicable; HRV: human live-attenuated vaccine containing G1P[8] strain; HBRV: human-bovine live-attenuated reassortant pentavalent vaccine; MenACWY-CRM: meningococcal serogroups A, C, W-135, and Y polysaccharide vaccine conjugated to diphtheria toxin CRM_197_; 4CMenB: recombinant, four-component meningococcal serogroup B vaccine adsorbed on aluminum hydroxide; MenAV: meningococcal serogroup A polysaccharide vaccine conjugated to tetanus toxoid; Hib-MenCY-TT: *Haemophilus influenzae* type b and meningococcal serogroups C and Y vaccine conjugated to tetanus toxoid; MenCC: meningococcal serogroup C polysaccharide vaccine conjugated to tetanus toxoid; MenC-CRM: Meningococcal serogroup C polysaccharide vaccine conjugated to diphtheria toxin CRM_197_; WoA: weeks of age; MoA: months of age; *World Health Organization Universal Trial Numbers.

## Safety

### Co-administration of RV and meningococcal vaccines compared to sequential administration

One randomized trial evaluated the co-administration of the HBRV vaccine and a tetanus toxoid conjugate MenCC vaccine (at 10–11 and 20–21 weeks of age, Group 1) compared to their sequential administration at alternating visits, at least four weeks apart (HBRV administered at 6–7 and 15–16 weeks of age, Group 2) in healthy infants aged 6–7 weeks.^29^ The third dose of HBRV was administered at 24–25 weeks of age in both groups. Both groups received additional routine vaccines (Supplementary Table S2). In the first 6 days after the first co-administered dose, proportion of participants with a solicited systemic adverse events (AEs) in Group 1 was 23.3% (diarrhea), 19.8% (vomiting), and 8.6% (fever), while 44.0% was assessed as related to HBRV (Table 3). In the 13-day period after their second dose, 42.6% of the children in Group 1 experienced at least one systemic AE related to HBRV. In Group 2, none of the systemic AEs were reported by >10% of the participants during the 13-day period following the second and third HBRV doses. Two serious adverse events (SAEs) occurred, one in each group: an episode of epilepsy of moderate intensity, starting 13 days after the second vaccination (Group 1) and a severe viral infection, starting 9 days after the third vaccination (Group 2). These events were considered not related to either study vaccine by the study investigators. One infant in each group experienced non-serious, mild hematochezia that was considered related to HBRV in Group 1 and to MenCC in Group 2.

**Table 3.** Systemic adverse events (%) related to co-administration of HBRV and MenCC or their sequential administration^28.^

	Group 1 (Co-administration)			Group 2 (Sequential administration)		
Systemic AEs (%)	Post-dose 1*	Post-dose 2*	Post-dose 3**	Post-dose 1**	Post-dose 2**	Post-dose 3**
Any (solicited/unsolicited) related to HBRV	65.5	42.6	<10	NR	<10	<10
Any solicited related to HBRV	44.0	27.0	NR	NR	NR	NR
Diarrhea	23.3^	13.0^	6.4	13.1	8.3	5.9
Vomiting	19.8^	10.4^	NR	5.7	NR	NR
Fever	8.6^	18.3^	NR	NR	NR	NR
Any unsolicited related to HBRV	45.7	24.3	NR	NR	NR	NR

*After co-administration of HBRV and MenCC vaccines. ** After sequential administration.

^ Unclear if related to meningococcal vaccine or HBRV vaccine. AE: Adverse event. NR: Not reported. HBRV: human-bovine live-attenuated reassortant pentavalent rotavirus vaccine. MenCC: MenC tetanus toxoid conjugate vaccine.

### Routine vaccination including co-administration of RV and meningococcal vaccines compared to routine vaccines including RV without meningococcal vaccines

Five phase III clinical studies investigated the co-administration of HRV or HBRV vaccines and conjugated meningococcal vaccines as part of routine immunization compared to vaccination without meningococcal vaccine, but including RV vaccines.^22,25-28^ The results could not be stratified for the AEs related to RV vaccines as other routine vaccines were also administered in all studies. We focused on diarrhea, vomiting, intussusception, change in eating habits, and fever (Table 4).

**Table 4.** SAE and a selection of solicited systemic adverse events (AEs) during concomitant administration of rotavirus vaccine and meningococcal vaccines plus other routine vaccines or individual administration of rotavirus vaccine plus other routine vaccines (n = 5 studies)

					SAE^#^ %		Fever % (severe %)		Vomiting % (severe %)		Diarrhea % (severe %)		Change in eating habits % (severe %)	
Reference	N	Rotavirus vaccine	Meningococcal vaccine	Primary schedule*	RV+ MV+ routine	RV + routine	RV+ MV+ routine	RV + routine	RV+ MV+ routine	RV + routine	RV+ MV+ routine	RV + routine	RV+ MV+ routine	RV + routine
Block 2016^27$^	733	Unknown	MenACWY-CRM	2, 4, 6**	4.6**;7.5	4.6	23.1**; 21.8	17.9	28.6**; 21.8	23.6	37.4**; 28.9	29.7	43.7**; 43.7	40.6
Klein 2012^28@^	1,364	HBRV	MenACWY-CRM	2, 4, 6	5.8	6.3	5	8	5 (0)	6 (<1)	7 (<1)	8 (<1)	15 (<1)	13 (1)
Klein 2017^22$^	600	HRV	Hib-MenCY-TT	2, 4, 6^%^	2.7	3.6	41.8	48.5	4.7	6.6	8.8	5.9	59.6	66.0
Tregnaghi 2014^26&^	3,033	HBRV	MenACWY-CRM	2, 4, 6^	8.5	9.5	5.0^; 13.3	11.3	8.3^(0.0); 14.2 (0.2)	12.5 (0.2)	14.0^ (0.3); 15.4 (0.2)	13.7 (0.2)	12.3^ (0.7); 17.1 (0.4)	17.2 (0.5)
Vesikari 2017^25@^	350	HBRV	MenCC	2, 3, 4	2.3	1.1	72.4 (1.1)	72.2 (0)	36.2 (2.3)	26.7 (1.1)	6.3^§^	4.0^§^	56.3 (1.7)	54.5 (2.8)

All studies provided routine vaccines concomitantly to combined rotavirus vaccine and meningococcal vaccine or rotavirus vaccine individually. When applicable, percentages of severe AEs are reported between brackets. N: number of participants included in the analysis. *At age in months, meningococcal vaccine schedule in bold. ** Two subgroups in total, in one subgroup MenACWY-CRM vaccine was provided at 2, 4, and 12 months of age and in the second group at 2, 4, 6, and 12 months of age. ^#^Data for SAE derived from clinicaltrials.gov except for Vesikari 2017. ^$^AE results at 7-day post-vaccination (primary and booster) derived from clinicaltrials.gov. ^&^ AE results at 7-day following first vaccination. ^@^AE results at 7-day post-primary vaccination. ^§^unsolicited AE results within 30-day of any vaccination. ^%^2-dose HRV vaccination, at 2 and 4 months. ^Two subgroups in total, in one subgroup MenACWY-CRM vaccine was provided at 2 and 6 months and in the second group at 2, 4 and 6 months of age. MV: meningococcal vaccine. RV: rotavirus vaccine. SAE: Serious adverse event. SAEs were recorded during the entire study period. Please see Table 1 footnote for definition of vaccines’ abbreviations.

One phase 3 study assessed the immunogenicity and safety of 3 + 1 doses of Hib-MenCY-TT vaccine when co-administered with routine vaccines including HRV.^22^ In the co-administration group, enrolled infants received 2 doses of HRV at 2 and 4 months of age and 3 primary doses of Hib-MenCY-TT at 2, 4, and 6 months of age and a booster dose at 12–15 months of age, along with other routine vaccines. In the control group, infants were administered with 2 HRV doses at 2 and 4 months of age, Hib vaccine without a meningococcal component at 2, 4, and 12–15 months of age, and other routine vaccines. Fever was reported by 41.8% of infants in the co-administration group and 48.5% in the control group; vomiting was reported by 4.7% and 6.6% of participants, diarrhea by 8.8% and 5.9%, change in eating habits by 59.6% and 66.0% of participants, respectively (Table 4). No cases of intussusception were reported. None of the reported SAEs was assessed as vaccination-related by the investigator.^22^

Three articles were identified on HBRV.^25,26,28^ Immunogenicity and safety of MenACWY-CRM, *Menveo* co-administered with routine vaccines including HBRV was assessed in a large phase 3 study conducted in the United States (US)^28^ and in Colombia and Argentina.^26^ After three infant doses of MenACWY-CRM and three HBRV doses at 2, 4, and 6 months of age in US infants, fever was reported by 5%, vomiting by 5%, diarrhea by 7%, and change in eating habits by 15% of study participants.^28^ When the same 3-dose vaccination schedule was evaluated in Colombia and Argentina, the incidence of solicited systemic AEs was 13.3% (fever), 14.2% (vomiting), 15.4% (diarrhea), and 17.1% (change in eating habits) after the first vaccination (at month 2).^26^ Co-administration of two primary doses of MenACWY-CRM (at 2 and 6 months of age) with 3 HBRV doses was also assessed in the Latin American study population. At month 2, fever, vomiting, diarrhea, and change of eating habits were reported by 5.0%, 8.3%, 14.0%, and 12.3% of participants, respectively.^26^ In both studies, comparable results were obtained in the control group who received routine vaccines without MenACWY-CRM. In the US population, three SAEs reported in the co-administration group were considered to be at least possibly related to vaccination: Kawasaki disease (29 days after the third dose), partial complex seizures (31 days after the second dose), and two episodes of febrile convulsions (8 and 29 days after the third dose).^28^

In the third article assessing the co-administration with HBRV, three doses of hexavalent combination vaccine (DTaP-IPV-HB-PRP-T, *Hexaxim*, at 2, 3, and 4 months of age) and two doses of MenCC vaccines (at 2 and 4 months of age) (Group 1) were compared to three doses of DTaP-IPV-HB-PRP-T vaccine without MenCC (Group 2). HBRV was administered in both groups at 2, 3, and 4 months of age together with 13-valent pneumococcal conjugate vaccine (PCV13, *Prevenar*, two doses at 2 and 4 months of age).^25^ Fever, vomiting, diarrhea, and change in eating habits were reported by 72.4%, 36.2%, 6.3%, and 56.3% of participants, respectively, in Group 1. Comparable values were obtained in Group 2 (Table 4). One participant in Group 1 experienced an SAE (fever ≤39.5°C on the day of first vaccination) that was considered related to study vaccines. No cases of intussusception were reported in any of the three articles.

The fifth study in this category compared the immunogenicity and safety of three (ACWY3 group, at 2, 4 and 12 months of age) or four doses (ACWY4 group, at 2, 4, 6 and 12 months of age) of MenACWY-CRM when co-administered with routine vaccines, including an RV vaccine.^27^ The control group received routine vaccines without MenACWY-CRM. Reactogenicity and safety profiles for all study groups were comparable, fever being reported by 23.1% (ACWY3), 21.8% (ACWY4), and 17.9% (control) of participants, vomiting by 28.6%, 21.8%, and 23.6%, diarrhea by 37.4%, 28.9%, and 29.7%, and change in eating habits by 43.7%, 43.7%, and 40.6% of participants, respectively.^27^ No cases of intussusception or vaccination-related SAEs were reported.

Additionally to the five aforementioned phase III clinical studies, safety data on the concomitant administration of 4CMenB and HRV were retrieved from a prospective surveillance study conducted among children up to 18 months of age who received 4CMenB as part of the NIP in the United Kingdom (UK).^30^ The study revealed no significant safety concerns within 20 months following 4CMenB introduction. Evaluation of immunization records for HRV vaccine collected before and after 4CMenB introduction suggested that 4CMenB reactogenicity did not affect compliance with the recommended vaccination schedule, including the administration of HRV.^30^

### Co-administration of RV and meningococcal vaccines, no comparative group

Four studies examined the co-administration of HRV or HBRV and meningococcal vaccines, however, no comparison was made with the administration of RV vaccines without meningococcal vaccines.^20,21,23,24^ Additional vaccines were also provided in all four studies as part of the local immunization program.

Two of the four studies were conducted in Spain to assess the immunogenicity and safety of several hexavalent and pentavalent combination vaccines administered in a mixed primary series concomitantly with other recommended vaccines.^20,24^ In a phase 3, single-arm study, the mixed 2-4-6-month schedule of hexavalent DTaP5-HB-IPV-Hib (*Vaxelis*) and pentavalent DTaP5-IPV-Hib (*Pediacel*) vaccines was co-administered with three HBRV doses (at 2, 4, 6 months of age) and MenCC and PCV13 vaccines (at 2 and 4 months of age). Solicited vaccine-related systemic AEs within 1–5 days following vaccinations at 2, 4, and 6 months of age were reported in 73.5%, 59.0%, and 42.7% of children, respectively. Of them, fever was reported in 4.9%, 6.2%, and 3.6%, vomiting in 12.7%, 8.1%, and 6.5%, and changing in eating habits in 36.6%, 22.9%, and 17.7% of participants.^20^

The second Spanish open-label study assessed the co-administration of HBRV (2, 4, 6 months of age), MenCC (2 months of age) and PCV13 (2, 4, 6 months of age) with DTaP-IPV-HB-PRP-T (*Hexaxim*) and DTaP-IPV//PRP-T (*Pentaxim*) (2, 4, 6 months of age). Any solicited systemic AE was reported in 97.4% of children, including 17.4% severe (grade 3) systemic AEs. Fever and vomiting were reported in 58.9% and 35.5% of children during the first 7 days after vaccination.^24^ There were no SAEs that were considered related to the study vaccines in either studies.

Although HBRV is recommended as a 3-dose primary vaccination series in infants from 6 weeks to 32 weeks of age, an additional HBRV dose co-administered with MenAV, measles, and yellow fever vaccines in 9–11-month-old infants was evaluated in Mali.^21^ Gastrointestinal illness was reported in 13.0% of the participants, including vomiting, diarrhea, or gastroenteritis, within 28 days after vaccination. No vaccination-related SAE and no cases of intussusception were reported.^21^

A review of the 4CMenB clinical development program reported results from a pooled sub-group analysis of two pivotal trials (5,515 participants) in which 303 infants had received at least one dose of RV vaccine (HRV or HBRV according to local recommendations) concomitantly with 4CMenB and other recommended vaccines.^23^ The study revealed comparable reactogenicity profiles in infants who did or did not receive RV vaccine. A systemic AE was reported for 80.5% of infants in the co-administration group and 75.3% of infants not receiving RV vaccines. Severe systemic reactions were recorded for 19.5% and 24.7% of participants, respectively. Frequency of fever cases was also comparable between group, high fever (≥39.5°C) being reported by 2.2–4.2% in the co-administration group and 2.6–4.5% in the group not receiving RV vaccines.^23^

Although letters to the editor were excluded from this systematic literature review (see Table 2), one of them was considered to contain relevant safety information and is described below. The letter reported solicited and unsolicited AEs from a study conducted in hospitalized preterm infants who received HRV vaccine either before or after the introduction of the 4CMenB vaccination into the NIP in the UK.^32^ Of 17 infants who completed the study, 8 received the 4CMenB vaccine. Overall, 4CMenB vaccine recipients were significantly more likely to have any temperature instability compared to infants receiving HRV without 4CMenB (50% versus 0%, *P* = .029). Decreased feeding and reduced activity were more common in the 4CMenB group, whereas irritability and crying occurred more frequently in the infants who did not receive the 4CMenB vaccine.

## Immunogenicity

Immunogenicity results were identified in five studies.^21,22,24,29,31^ Geometric mean concentrations and titers are summarized in Table 5, seroconversion and seroresponse rates are presented in Table 6. Seroconversion rate was defined as the percentage of infants with anti-RV immunoglobulin A and G (IgA and IgG) ≥20 U/mL post-vaccination (measured by enzyme-linked immunosorbent assay) who had antibody titer below this threshold pre-vaccination. Seroresponse rates were defined as at least threefold increase in IgA response from pre- to post-vaccination. Two studies did not present data for a comparator group with individual RV vaccination.^21,24^

**Table 5.** Rotavirus immunogenicity measured as geometric mean titer or geometric mean concentration (n = 4 studies)

Reference	N	Rotavirus vaccine	Meningococcal vaccine	Schedule*	Measured at day; post-dose	Parameter	GMT or GMC (95%CI)	
							Co-administration	Sequential^%^/individual RV administration
Klein 2019^22^	316	HRV	Hib-MenCY-TT	2, 4, 6^#^	2 M; 2	IgA antibody titer	138.9 (104.0–185.5)^	115.0 (87.5–151.0)^
Vesikari 2011^29^	202	HBRV	MenCC	2, 4, 6	42; 3	IgA antibody titer	290.6 (215.1–392.5)	363.1 (290.3–454.2) ^%^
	202	HBRV	MenCC	2, 4, 6	42; 3	SNA response to G1	187.2 (148.8–235.5)	211.4 (168.6–264.9) ^%^
						SNA response to G2	41.9 (33.7–52.2)	44.3 (35.6–55.1) ^%^
						SNA response to G3	24.2 (18.8–31.1)	25.5 (19.8–32.9) ^%^
						SNA response to G4	64.7 (51.1–82.0)	76.4 (59.8–97.5) ^%^
						SNA response to P1A[8]	111.2 (88.3–140.0)	124.3 (99.1–156.0) ^%^
Haidara 2018^21^	292	HBRV	MenAV	Booster dose at 9 to 11	28	IgA antibody titer	118.4 (90.9–154.3)^	NA
	292	HBRV	MenAV	Booster dose at 9 to 11	28	IgG antibody titer	363.6 (293.6–450.4)^	NA
Martinon-Torres 2019^24^	263	HBRV	MenCC	2, 4, 6	1 M; 3	IgG antibody titer	279.0 (214.0–362.0)	NA

All studies provided routine vaccines concomitantly to combined rotavirus vaccine and meningococcal vaccine or rotavirus vaccine individually. *At age in months, meningococcal vaccine schedule in bold. ^GMC: Geometric mean concentration. ^#^2-dose HRV vaccination, at 2 and 4 months. ^%^Meningococcal vaccine and rotavirus vaccine provided sequentially. GMT: Geometric mean titer. N: number of participants included in the analysis. M: Month. NA: Not applicable. IgA: immunoglobulin A. SNA: serotype-specific rotavirus neutralizing antibody.

**Table 6.** Seroconversion and seroresponse rates following rotavirus and meningococcal vaccination (n = 5 studies)

Reference	N	Rotavirus vaccine	Meningococcal vaccine	Schedule*	Measured at day; post-dose	Parameter	Levels	Seroconversion or seroresponse (%) (95%CI)	
								Co-administration	Sequential^%^/individual RV administration
Klein 2019^22^	316	HRV	Hib-MenCY-TT	2, 4, 6^#^	2 M; 2	IgA	≥20 U/ml (overall)	81.3 (74.2–87.1)	80.1 (73.1–86.0)
Vesikari 2010^31^	1,216	HRV	MenC-CRM	2, 4	1-2 M; 2	IgA	≥20 U/ml (and <20 U/ml at baseline)	85.5 (79.6–90.2)^	94.6 (90.0–97.5)^ 92.3 (64.0–99.8)^ 82.1 (75.1–87.7)^ 84.3 (74.7–91.4)^ 84.6 (78.5–89.5)^
Vesikari 2011^29^	202	HBRV	MenCC	2, 4, 6	42; 3	IgA	≥3 fold increase ^$^	96.9 (91.3–99.4)	98.1 (93.2–99.8) ^%^
	202	HBRV	MenCC	2, 4, 6	42; 3	G1**	≥3 fold increase^$^	57.1 (46.7–67.1)	63.5 (53.4–72.7) ^%^
						G2**	≥3 fold increase^$^	33.7 (24.4–43.9)	38.5 (29.1–48.5) ^%^
						G3**	≥3 fold increase^$^	33.7 (24.4–43.9)	37.5 (28.2–47.5) ^%^
						G4**	≥3 fold increase^$^	44.9 (34.8–55.3)	46.2 (36.3–56.2) ^%^
						P1A[8]**	≥3 fold increase^$^	32.7 (23.5–42.9)	40.4 (30.9–50.5) ^%^
Haidara 2018^21^	292	HBRV	MenAV	Booster dose at 9 to 11	28	IgA	≥20 U/ml (and <20 U/ml at baseline)	56.9 (49.2–64.5)	NA
							≥20 U/ml (overall)	74.7 (69.7–79.6)	NA
							≥3 fold increase^$^	44.9 (39.2–50.6)	NA
	292	HBRV	MenAV	Booster dose at 9 to 11	28	IgG	≥20 U/ml (and <20 U/ml at baseline)	83.5 (75.9–91.1)	NA
							≥20 U/ml (overall)	93.9 (91.1–96.6)	NA
							≥3 fold increase^$^	57.3 (51.7–63.0)	NA
Martinon-Torres 2019^24^	263	HBRV	MenCC	2, 4, 6	1 M; 3	IgA	≥20 U/ml (and <20 U/ml at baseline)	88.4 (82.1–93.1)	NA
	263	HBRV	MenCC	2, 4, 6	1 M; 3	IgG	≥20 U/ml (overall)	90.8 (85.6–94.5)	NA

NB. All studies provided routine vaccines concomitantly to combined rotavirus vaccine and meningococcal vaccine or rotavirus vaccine individually. Seroconversion rate was defined as percentage of infants with anti-RV immunoglobulin A and G (IgA and IgG) ≥20 U/mL (measured by enzyme-linked immunosorbent assay) post-vaccination who had antibody titer below the threshold pre-vaccination. Seroresponse rates were defined as at least threefold increase in IgA response from pre- to post-vaccination. *At age in months, meningococcal vaccine schedule in bold. ** SNA response to rotavirus genotype. ^#^2-dose HRV vaccination, at 2 and 4 months. **^%^** meningococcal vaccine and rotavirus vaccine provided sequentially. **^$^** ≥3 fold increase from pre- (baseline) to postvaccination. ^Co-administration results are for Spain. Results of control group are the results for the countries: Finland, Italy, Germany, France, and Czech Republic, respectively, where no meningococcal vaccine was administered. N: number of participants included in the analysis. M: Month; NA: Not applicable; SNA: serotype-specific rotavirus neutralizing antibody.

In a phase 3b, placebo-controlled study conducted across six European countries (the Czech Republic, Finland, France, Germany, Italy, and Spain), HRV or placebo were co-administered with routine vaccines recommended in the respective countries. Immunogenicity results were available from 794 infants in the HRV group and 422 infants in the placebo group. Spain was the only country administering a meningococcal vaccine (MenC-CRM, *Meningitec*) during routine immunizations, thus, descriptive results for Spain compared to other countries are provided. The seroconversion rate was 85.5% after co-administration of HRV and MenC-CRM in Spain and ranged from 82.1% to 94.6% after administration of HRV in other countries (Table 6).^31^

Another study conducted in the US demonstrated non-inferiority of two primary doses of HRV co-administered with Hib-MenCY-TT and other recommended vaccines compared to similar vaccination series without meningococcal vaccine in terms of anti-RV IgA antibody concentrations.^22^ Two months post-vaccination, anti-RV IgA concentrations ≥20 U/mL were detected in 81.3% of infants in the co-administration group and 80.1% in those receiving HRV without meningococcal vaccine^22^ (Table 6).

Immunogenicity data for co-administration of HBRV and MenCC were retrieved from two studies.^24,29^ Seroconversion rates were 88.4% in the co-administration group in one study (with no comparative group)^24^ while in the second study, seroresponse rates were 96.9% in the co-administration group and 98.1% in the group receiving the vaccines sequentially^29^ (Table 6).

The study evaluating the immunogenicity of an additional HBRV dose co-administered with MenAV vaccine in children who completed the recommended 3-dose primary HBRV vaccination schedule reported seroconversion rates for 56.9% (anti-RV IgA) and 83.5% (anti-RV IgG) of participants and seroresponse rates for 44.9% and 57.3%, respectively (Table 6).^21^

## Efficacy

No studies reporting efficacy results of RV vaccination and meningococcal vaccination were identified by this systematic review.

## Discussion

This systematic review aimed to retrieve and summarize the currently available evidence on the safety profile, immunogenicity, and efficacy of RV vaccines when co-administered with available meningococcal vaccines in infants. The limited number of studies presenting a comparative evaluation of RV vaccines when administered with or without meningococcal vaccines, as well as the heterogeneity in study designs and local immunization programs led to a descriptive analysis of the data without applying meta-analysis methods. Other routine injectable vaccines were also administered in all studies, thus no direct comparison between RV vaccine alone and RV and meningococcal vaccines together could be made. However, the concomitant administration of other routine vaccines is representative of the current vaccination practice. The quality of studies included was generally acceptable. In most of the studies, investigators were not blinded for the study allocation groups, which could be a limitation in the perspective of the assessment of AEs. Our search identified only one study with immunogenicity data in low- and middle-income countries. This is considered another limitation since the disease burden of both RV and meningitis is high in this setting.^33^

Safety analysis was mainly focused on AEs that might be associated with RV vaccination, such as fever, diarrhea, vomiting, change in eating habits, and intussusception, and found no new safety concerns upon co-administration with meningococcal vaccines. In the only study comparing directly the co-administration of HBRV and MenCC vaccines with their sequential administration, the incidence of gastrointestinal symptoms tended to be lower in the sequential group than in the co-administration group.^29^ Although the difference was not considered clinically significant, the authors point out that the convenience of co-administration of HBRV and MenCC may outweigh the slight increase in risk of mild diarrhea and mild vomiting associated with co-administration.^29^ In studies comparing routine co-administration of RV and meningococcal vaccines with vaccination schedules without meningococcal vaccines, similar rates of solicited systemic AEs were found.^22,25-28,30^ Studies reporting safety data for co-administration of RV and 4CMenB vaccines were mainly focused on 4CMenB-related safety aspects, but reactogenicity of 4CMenB was not impacted by the concomitant administration of RV vaccines, supporting their concomitant use in routine practice.^23,30^ Following the introduction of 4CMenB to the NIP in the UK in 2015, no significant safety concerns were observed.^30^

There is a theoretical concern that the co-administration of multiple antigens may lead to immune interference.^34^ The reviewed literature revealed that anti-RV IgA response rates were comparable between groups of children receiving RV and meningococcal vaccines versus those receiving RV vaccines alone or subsequently, regardless of the administration of other routine vaccines.^22,29,31^ Moreover, serotype-specific rotavirus neutralizing antibody response rates were similar for children receiving HBRV and MenCC either concomitantly or separately.^29^ This suggests the absence of immune interference, but might also be explained by the differences in the route of administration (oral for RV vaccines versus intramuscular for meningococcal vaccines) along with vaccine type and composition.^35,36^ Only one study reported immunogenicity data for 4CMenB when co-administered with RV. Immune responses of 4CMenB did not appear to interfere with the immunogenicity of any of the vaccines tested, including RV.^23^

Oral poliovirus vaccine (OPV), the only oral vaccine commonly co-administered with RV vaccine, may slightly reduce the immune response to RV vaccine, but clinical protection against severe RVGE is maintained.^37,38^ Since OPV was not administered in any of the studies included in this review, we could not evaluate the co-administration of RV vaccines, meningococcal vaccines, and OPV.

Worldwide, several national and international agencies recommend the co-administration of RV vaccines with other infant vaccines against diphtheria, tetanus, pertussis, Hib, poliovirus, Hepatitis B, Pneumococcal, and Meningococcal disease.^2,16,17^ Co-administration of vaccines could contribute to better vaccine coverage and could reduce medical visits and associated costs. It also helps to provide protection for children during the vulnerable early months of their lives.

**Figure 2.**
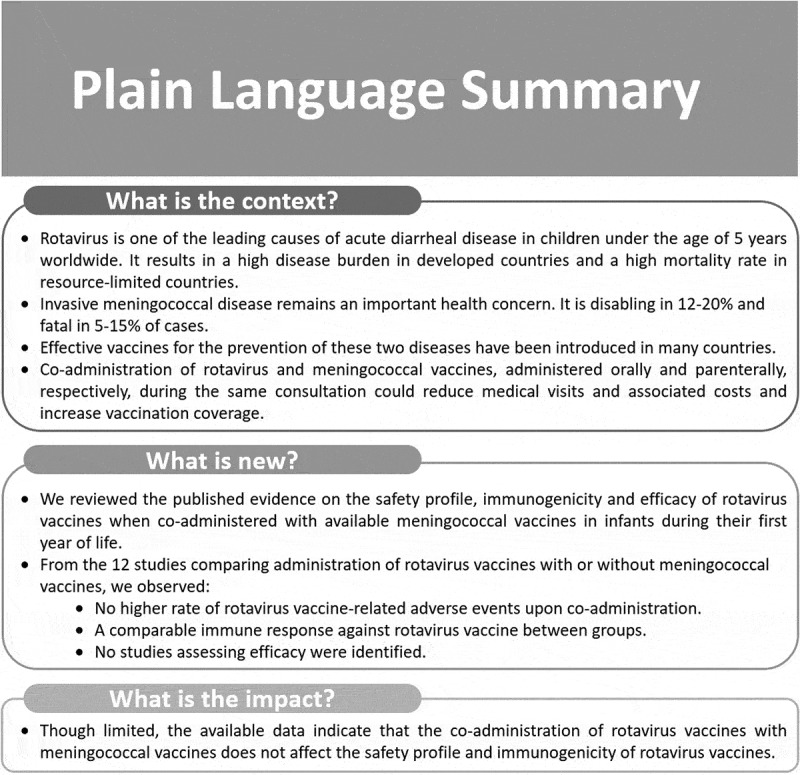
Plain language summary

A summary contextualizing the results and their potential clinical relevance is provided in Figure 2 to assist communications to the parents.

## Conclusions

Despite the limited data, co-administration of RV and meningococcal vaccines does not appear to interfere with the safety or immunogenicity of the two globally available RV vaccines.

## Supplementary Material

Supplemental Material

## Trademark statement

*Rotarix, Menjugate, Menveo, MenHibrix*, and *Bexsero* are trademarks owned by the GSK group of companies. *RotaTeq* is a registered trademark of Merck&Co, Inc. *Rotavac* is a registered trademark of Bharat Biotech. *Rotasiil* and *MenAfriVac* are registered trademarks of Serum Institute of India Ltd. *NeisVac-C* is a registered trademark of Baxter International Inc. *Meningitec* is a registered trademark of Nuron Biotech. *Menactra* is a registered trademark of Sanofi.
