# Supplementary material for: Systematic literature review on the safety and immunogenicity of rotavirus vaccines when co-administered with meningococcal vaccines
Source: Hum Vaccin Immunother. 2020 Apr 16;16(11):2861–72. doi: 10.1080/21645515.2020.1739485 (PMC7746238; doi:10.1080/21645515.2020.1739485)
Supplement: Supplemental Material [file KHVI_A_1739485_SM7466.docx]

# Supplemental material

## Table S1. Strings used in the literature search

|  | **PubMed** | | **Embase** |
| --- | --- | --- | --- |
| **Search strings** | *#1. Meningococcal disease*  "Meningococcal Infections"[Mesh] OR "Meningitis, meningococcal"[Mesh] OR Neisseria meningitidis[Mesh] OR meningococc*[tw] OR Neisseria meningitid*[tw]  *#2. Rotavirus infection*  "Rotavirus"[Mesh] OR "Rotavirus Infections"[Mesh] OR rotavir*[tw]  *#3. Vaccination*  "Vaccination"[Mesh] OR "Antibodies"[Mesh] OR “Immunization”[Mesh] OR vaccin*[tw] OR immuniz*[tw] OR immunis*[tw] OR immune[tw] OR immunity[tw] OR immunology[tw] OR antibod*[tw]  *#4. Meningococcal vaccines*  "4CMenB vaccine" [Supplementary Concept] OR "MenACWY-CRM vaccine" [Supplementary Concept] OR "Meningococcal Vaccines"[Mesh] OR Bexsero*[tw] OR Nimenrix*[tw] OR Menveo*[tw] OR Neisvac-C*[tw] OR meningococcal vaccine*[tw] OR MenACWY*[tw] OR MenACWY-CRM[tw] OR MenACWY-TT[tw] OR MenB-4C[tw] OR MenC-CRM[tw] OR Menjugate*[tw] OR MenC-TT[tw] OR MenC-TT/Hi-TT[tw] OR Menitorix*[tw]  *#5. Rotavirus vaccines*  "RIX4414 vaccine"[Supplementary Concept] OR "Rotavirus Vaccines"[Mesh] OR "rhesus rotavirus vaccine"[Supplementary Concept] OR "RotaTeq"[Supplementary Concept] OR "VP3 protein, Rotavirus"[Supplementary Concept] OR "VP2 protein, Rotavirus"[Supplementary Concept] OR "rotavirus vaccine 89-12"[Supplementary Concept] OR "WC3 rotavirus vaccine"[Supplementary Concept] OR "RV3 rotavirus vaccine"[Supplementary Concept] OR "VP1 protein, Rotavirus"[Supplementary Concept] OR "VP6 protein, Rotavirus"[Supplementary Concept] OR "VP7 protein, Rotavirus"[Supplementary Concept] OR "VP4 protein, Rotavirus"[Supplementary Concept]) OR rotarix*[tw] OR rotateq*[tw] OR wc3[tw] OR RIX4414[tw] OR RV5[tw] OR RV1[tw]  *#6.* *Coadministration*  "Vaccines/administration and dosage"[Majr] OR "Vaccination/administration and dosage”[Majr] OR coadministration[tw] OR co-administration[tw] OR coadminister*[tw] co-administer*[tw] OR combined[tw] OR concomitant*[tw] OR concurrent*[tw] OR “administered with”[tw] OR “administered concomitantly”[tw] OR “administered together”[tw] OR “administered in combination”[tw] OR “administered combined”[tw] OR “routine immunisation”[tw] OR “routine immunization”[tw] OR schedule[tw] | | *#1. Meningococcal disease*  ‘meningococcosis’/exp OR ‘meningitis’:ti,ab OR ‘neisseria meningitidis’:ti,ab OR meningococc*:ti,ab OR ‘neisseria meningitidi*’:ti,ab  *#2. Rotavirus infection*  ‘rotavirus’/exp OR rotavir*:ti,ab  *#3. Vaccination*  ‘vaccination’/exp OR ‘immunization’/exp OR vaccin*:ti,ab OR immuniz*:ti,ab OR immunis*:ti,ab OR immune:ti,ab OR immunity:ti,ab OR immunology:ti,ab OR antibod*:ti,ab  *#4. Meningococcal vaccines*  ‘menacwy-crm vaccine’/exp OR ‘4cmenb vaccine’/exp OR ‘meningococcus vaccine’/exp OR ‘meningococcal vaccin*’:ti,ab OR bexsero*:ti,ab OR nimenrix*:ti,ab OR menveo*:ti,ab OR neisvac-c*:ti,ab OR menacwy*:ti,ab OR ‘menacwy-crm’:ti,ab OR ‘menacwy-tt’:ti,ab OR ‘menb-4c’:ti,ab OR ‘menc-crm’:ti,ab OR menjugate*:ti,ab OR ‘menc-tt*’:ti,ab OR menitorix*:ti,ab  *#5. Rotavirus vaccines*  rotarix*:ti,ab OR rotateq*:ti,ab OR rotavirus) AND vaccin*:ti,ab OR rhesus AND vaccin*:ti,ab OR rhesus:ti,ab OR protein) AND vp3:ti,ab OR protein) AND vp2:ti,ab OR protein) AND vp1:ti,ab OR '89 12':ti,ab OR wc3:ti,ab OR rv3:ti,ab OR rv5:ti,ab OR rv1:ti, ab OR (protein AND vp6:ti,ab OR (protein AND vp7:ti,ab) OR (protein AND vp4:ti,ab) OR rix4414:ti,ab)  *#6.* *Coadministration*  Administrat*:ti,ab OR dosage*:ti,ab OR coadministration:ti,ab OR ‘co-administration’:ti,ab OR coadminister*:ti,ab OR ‘co-administer*’:ti,ab OR combined:ti,ab OR concomitant*:ti,ab OR concurrent*:ti,ab OR ‘administered with’:ti,ab OR ‘administered concomitantly’:ti,ab OR ‘administered together’:ti,ab OR ‘administered in combination’:ti,ab OR ‘administered combined’:ti,ab OR ‘routine immunisation’:ti,ab OR ‘routine immunization’:ti,ab OR schedule:ti,ab OR ((vaccin*:ti,ab OR administrat*:ti,ab) AND dosage:ti,ab) |
| **Time limit** | Publication date: 01 January 2000 – 04 January 2019 | | 2000:py OR 2001:py OR 2002:py OR 2003:py OR 2004:py OR 2005:py OR 2006:py OR 2007:py OR 2008:py OR 2009:py OR 2010:py OR 2011:py OR 2012:py OR 2013:py OR 2014:py OR 2015:py OR 2016:py OR 2017:py OR 2018:py OR 2019:py AND [embase]/lim NOT ([embase]/lim AND [medline]/lim) |
| **String combination** | Rotavirus vaccination and coadministration: ((#2 AND #3) OR #5) AND #6  Meningococcal vaccination and coadministration: ((#1 AND #3) OR #4) AND #6 | | |
| **COCHRANE LIBRARY** | | | |
| - Rotavirus vaccin* AND coadministration OR co-administration - Rotavirus vaccin* AND mening* vaccin* AND concomitant | | | |
| **LILACS** | | | |
| **Rotavirus vaccination** | | (tw:(rotavir*)) OR ((tw:(rotarix*)) OR (tw:(rotateq*)) OR (tw:(RIX4414)) OR (tw:(RV1)) OR (tw:(RV5))) AND ((tw:(coadministration)) OR (tw:(co-administration)) OR (tw:(coadminister*)) OR (tw:(co-administer*)) OR (tw:(combined)) OR (tw:(concomitant*)) OR (tw:(concurrent*)) OR (tw:("routine immunization")) OR (tw:(schedule)) OR (tw:("routine immunisation")))  Limits: LILACS database AND year 2000-2019 | |
| **Meningococcal vaccination** | | (tw:("Meningococcal Infections)) OR (tw:("Meningitis)) OR (tw:(meningococcal)) OR (tw:(Neisseria meningitidis)) OR (tw:(meningococc*)) OR (tw:(meningitid*)) OR ((tw:(Bexsero*)) OR (tw: (Nimenrix*)) OR (tw:(Menveo*)) OR (tw:(Neisvac-C*)) OR (tw:(meningococcal vaccine*)) OR (tw:(MenACWY*)) OR (tw:(MenACWY-CRM)) OR (tw:(MenACWY-TT)) OR (tw:(MenB-4C)) OR (tw:(MenC-CRM)) OR (tw:(Menjugate)) OR (tw:(MenC-TT)) OR (tw:(MenC-TT/Hi-TT)) OR (tw:(Menitorix*))) AND ((tw:(coadministration)) OR (tw:(co-administration)) OR (tw:(coadminister*)) OR (tw:(co-administer*)) OR (tw:(combined)) OR (tw:(concomitant*)) OR (tw:(concurrent*)) OR (tw:("routine immunization")) OR (tw:(schedule)) OR (tw:("routine immunisation")))  Limits: LILACS database AND articles published from 2000 onwards. | |
| **GREY LITERATURE SEARCH (WHO, CDC, PAHO, ECDC websites, Google)** | | | |
| - “Rotavirus vaccination AND coadministration” - “Rotavirus vaccination AND concomitant” - “Rotavirus AND meningococcal vaccines AND coadministration” - “Meningococcal vaccination AND coadministration” | | | |

## Table S2. Additional vaccines co-administered with rotavirus and meningococcal vaccines or rotavirus vaccines, at age in months

| **Author, year** | **(Sub)group** | **2 months** | **3 months** | **4 months** | **6 months or >6 months -≤12 months** |
| --- | --- | --- | --- | --- | --- |
| Block, 2016^[2](#_ENREF_25)7^ | Different per region* | PCV13, DTaP-IPV-Hib, HBV, MMRV or MMR and varicella vaccine* | * | * | * |
| Bryan, 2018^30^ | All | DTaP-IPV-Hib and PCV13** | NA | DTaP-IPV-Hib and PCV13*** | NA |
| Haidara, 2018^[21](#_ENREF_21)^ | All | NA | NA | NA | Measles and yellow fever booster dose |
| Klein, 2012^[28](#_ENREF_28)^ | All | DTaP-HBV-IPV, Hib-TT, PCV7 |  | DTaP-HBV-IPV, Hib-TT, PCV7 | DTaP-HBV-IPV, Hib-TT, PCV7 |
| Klein, 2019^[2](#_ENREF_20)2^ | Co-administration of RV and MV | Hib, PCV13 and DTaP-HBV-IPV | NA | Hib, PCV13 and DTaP-HBV-IPV | Hib, PCV13 and DTaP-HBV-IPV |
|  | Individual administration of RV | Hib-OMP, PCV13 and DTaP-HBV-IPV | NA | Hib-OMP, PCV13 and DTaP-HBV-IPV | Hib-OMP^#^, PCV13 and DTaP-HBV-IPV, HAV^#^ |
| Martinon-Torres, 2017^[2](#_ENREF_24)0^ | All | DTaP5-HB-IPV-Hib and PCV13 | NA | DTaP5-IPV-Hib and PCV13 | DTaP5-HB and IPV-Hib |
| Martinon-Torres, 2019^[24](#_ENREF_19)^ | All | DTaP-IPV-HB-PRP-T and PCV13 | NA | DTaP-IPV/PRP and PCV13 | DTaP-IPV-HB-PRP-T and PCV13 (optionally) |
| Tregnaghi, 2014^[26](#_ENREF_26)^ | All | DTaP-HBV-IPV, Hib-TT, PCV7 | NA | DTaP-HBV-IPV, Hib-TT, PCV7 | DTaP-HBV-IPV, Hib-TT, PCV7 and MMRV, PCV7 and HAV |
| Vesikari, 2010^[3](#_ENREF_30)1^ | Different per country | DTaP-HBV-IPV-Hib (All countries) and PCV7 (France and Germany only) | ^ | DTaP-HBV-IPV-Hib or DTaP-IPV-Hib (France) and PCV7 (France and Germany) | ^ |
| Vesikari, 2011^[29](#_ENREF_29)^ | All | DTaP-IPV-Hib | NA | DTaP-IPV-Hib | NA |
| Vesikari, 2017^[2](#_ENREF_23)5^ | All | DTaP-IPV-HB-PRP-T and PCV13 | DTaP-IPV-HB-PRP-T | DTaP-IPV-HB-PRP-T and  PCV13 | NA |

* According to the recommended schedule in the respective regions. **At 8 weeks of age. ***At 16 weeks of age. ^#^at 12-15 months of age. ^Schedule differed per country; France and Germany at 2 and 3 months of age; Czech Republic at 3 and 4 months. Spain at 2 and 4 months; Italy and Finland at 3 and 5 months. Additional vaccines (dose 3) were provided at 4, 5 and 6 months, not clear which vaccines. DTaP: Diphtheria, tetanus acellular pertussis. HAV: Hepatitis A Vaccine. HB: Hepatitis B. HBV: Hepatitis B vaccine Hib: *Haemophilus influenzae* type b. IPV: Inactivated polio vaccine. MMR: Measles, mumps, rubella vaccine. MMRV: Measles, mumps, rubella and varicella vaccine. MV: meningococcal vaccine. NA: not available. OMP: Outer membrane proteins. PCV7: Prevnar 7, pneumococcal conjugate vaccine. PCV13: Prevnar 13, pneumococcal conjugate vaccine. PRP-T: Polyribosylribitol phosphate conjugated to tetanus protein. RV: Rotavirus vaccine. TT: Tetanus toxoid.
